# The role of early social play behaviors and language skills for shy children’s later internalizing difficulties in school

**DOI:** 10.3389/fpsyt.2023.1120109

**Published:** 2023-03-01

**Authors:** Silje Baardstu, Stefania Sette, Ragnhild Eek Brandlistuen, Mari Vaage Wang

**Affiliations:** ^1^Department of Childhood and Families, Norwegian Institute of Public Health, Oslo, Norway; ^2^Department of Developmental and Social Psychology, Sapienza University, Rome, Italy; ^3^Department of Child Health and Development, Norwegian Institute of Public Health, Oslo, Norway

**Keywords:** MoBa, shyness, adjustment, longitudinal, risk and protective factors, anxiety, depression, language

## Abstract

Research has demonstrated links from early childhood shyness to socioemotional problems later in life. This longitudinal study explored the role of early social play behaviors and language skills in the associations between childhood shyness and later internalizing and language difficulties in school. Participants were *N* = 7,447 children (50.1% girls) from the Norwegian Mother, Father, and Child Cohort Study (MoBa). Latent direct, indirect, and interaction path analyses were performed within a structural equation framework. Results showed that mother-rated childhood shyness from age 18 months to age five years was associated with mother-rated internalizing difficulties and language problems at age eight years. Lower levels of teacher-reported social play behaviors and poorer language skills in preschool increased the risk of later anxiety problems among shy children, whereas higher levels of language competencies and social play behaviors buffered against later anxiety problems. The study identifies some of the early risk and protective factors that may influence shy children’s socio-emotional functioning and adjustment.

## 1. Introduction

Shyness is a temperament trait that is characterized by wariness, self-consciousness, and reticence in the face of social novelty and/or situations of perceived social evaluation (1). An extensive body of studies have demonstrated how early shyness confer increased risk for both concurrent and prospective difficulties in several developmental domains, and particularly in the areas of socio-communicative skills and socioemotional adjustment (2, 3). However, there is growing recognition for the notion that there is heterogeneity in these observed outcomes [i.e., (4–7)]. In other words, not all shy children are destined to experience or develop such difficulties. This notion elucidates the importance of identifying early risk and protective factors that may influence shy children’s developmental course, as this would allow for greater precision in understanding individual differences in the adjustment and functioning of shy children.

Several factors that may influence shy children’s adjustment have been suggested in the past decades, including the role of *biological* factors, such as cortisol levels, *individual* factors, such as regulatory functions [i.e., attention bias and inhibitory control; (8–12)], and *environmental* factors, such as parental socialization practices and the quality of relationships with peers and others [for an overview, see (13)]. Some studies have also demonstrated the protective (i.e., moderating) role of language skills and social competencies for shy children’s social and emotional functioning (2, 14–17).

However, most of this research has mainly been cross-sectional and using small samples of relatively young children (i.e., toddlers, preschoolers). For this reason, there is a need for more longitudinal studies that may expand our knowledge about the long-term nature of associations between shyness, social behaviors, language skills, and internalizing difficulties from early childhood and into school age, including indirect pathways as well as moderating processes. Thus, using longitudinal data from more than 7,000 children followed from 18 months to age 11 years, the purpose of the present study was twofold: the first aim was to examine the long-term prospective links between early childhood shyness and later internalizing and language problems in school, and the second aim was to explore the mediating and/or moderating role of children’s early social play behaviors and language competencies, as reported by early childhood and education care (ECEC) teachers, in these links.

Temperamental shyness overlaps conceptually with other related constructs, such as anxious solitude, behavioral inhibition, and social reticence. For instance, all these constructs share an underlying core related to social fear, anxiety, and wariness, and they also display relatively similar patterns of associations with adjustment difficulties [see (13), for an overview]. In this study, we conceptualize shyness as a temperament trait that is characterized by wariness, anxiety, and discomfort in response to social novelty and/or self-consciousness in situations of perceived social evaluation (1, 18). This discomfort mainly derives from the interpersonal nature of situations and often elicits inhibited and awkward behaviors, including a desire to withdraw from social interactions (19).

Over the past decades, much research has uncovered the behavioral and psychosocial correlates of temperamental shyness in childhood [for a review, see Rubin et al. (20)]. On a behavioral level, shyness may manifest as avoidant, withdrawn, awkward, and inhibited behaviors (e.g., freezing behaviors, watching other children playing but not joining in) during interactions with peers or in unfamiliar social situations (21, 22). Psychosocially, shy children tend to experience lower self-esteem, are perceived to have lower social competence, and have higher anxiety levels compared to non-shy children (1, 23).

Many shy children may also experience an approach-avoidance conflict in which their desire for social interaction (i.e., social approach) is simultaneously inhibited by social fear and anxiety (i.e., social avoidance) (19, 24). This motivational conflict could perhaps explain why shy children often talk less and are less likely to initiate and participate in social interactions with peers compared to more outgoing children (2). Consequently, there is a concern that shy children may have fewer opportunities to practice and develop social and communicative skills and competencies, and that they may also develop feelings of low self-worth and poor self-esteem if they often experience failure in their social engagement with peers (25, 26). In this sense, there is a general conception that shyness is associated with increased risk for poor socio-emotional functioning, including peer problems and loneliness, which, in turn, may increase their risk for emotional difficulties, such as social anxiety and depression [e.g., (27, 28)].

A growing literature has underlined that shyness is concurrently related to emotional difficulties, such as social anxiety and depression, throughout development [i.e., (29, 30)]. Longitudinal studies have also confirmed such links. For instance, Poole et al. (31), across three laboratory visits in early-to-middle childhood, found two trajectories of shyness, including a high-stable class and a low-stable class. Results revealed that teachers and parents perceived children in high-stable classes as more socially anxious than children in the low-stable class. Gender differences were also found in the high-stable class, with boys displaying more depressive symptoms than girls. Furthermore, Karevold et al. (3), following a sample of 921 Norwegian children from 18 months to 12.5 years, reported that mother-reported shyness at age four was a predictor of parent and self-reported anxiety and parent-reported depression (the latter with a lower effect size) at 12.5 years. Similarly, Bohlin and Hagekull (32), in a sample of 100 participants, found that parent-reported shyness in infancy was a significant predictor of social anxiety and depression at 21 years as reported by participants themselves; however, the association between early shyness and later depression was no longer significant when the analysis controlled for social anxiety at age 21.

Overall, these findings from both concurrent and longitudinal studies are in line with a recent metanalysis which concluded that behavioral inhibition (a construct conceptually similar to shyness) in early childhood represents one of the main risk factors of subsequent anxiety, and especially social anxiety disorders (28). Yet, although the concurrent and longitudinal associations between shyness and internalizing problems have been empirically demonstrated, the roles of possible risk and protective factors that may exacerbate or mitigate such links over time are less clear (28).

In addition to emotional problems, research has also consistently shown shyness to be associated with language and socio-communicative difficulties, including less complex language and poorer expressive, pragmatic, and receptive language skills [for an overview, see Coplan et al. (2)]. Shy children are also found to speak less than less shy children in familiar and unfamiliar settings (33) and to show a skill delay in the social use of language (34).

Several processes behind the shyness and language problems link have been suggested, as summed up by Coplan and Evans (35). For instance, as language is learned through active participation, shy children may be less linguistically competent because their withdrawn demeanor and restricted verbal participation in social settings hampers their opportunities to acquire, practice, and develop language skills (i.e., “competence deficit”). Others suggest that shy children’s poorer verbal abilities may rather reflect a “performance deficit” in which shy children’s underlying social anxiety and wariness may act to inhibit these children’s propensity to speak and respond in social settings (36). Support for this latter view comes from research showing that more shy children often score lower on tests of expressive language than on tests of receptive language (37).

Despite the vast body of studies linking shyness with adjustment difficulties and poor social and communicative functioning, it is clearly not the case that all shy children experience such difficulties. In this respect, a growing body of research has suggested several risk and protective factors that may influence shy children’s developmental course, including biological factors (i.e., cortisol levels) and environmental factors (i.e., parental socialization practices, the quality of relationships with others) [for an overview, see Coplan et al. (13)].

A small body of studies has also explored the role of individual factors for shy children’s socio-emotional functioning. For instance, studies have reported that the association between shyness and internalizing problems is lower among shy children with higher levels of temperamental activity or greater sports participation (38, 39). Furthermore, shy preschool-children with higher emotion-related competencies (i.e., ability to recognize the emotions of others) are found to show better social and emotional adjustment (i.e., less anxious-withdrawal and peer rejection) compared to shy children with lower levels of emotion recognition (30). Moreover, greater inhibitory control, a component of temperamental effortful control, is found to increase shy children’s risk of negative adjustment, including heightened anxiety problems in kindergarten (8), higher levels of social anxiety, as well as less prosocial behaviors and more problematic peer interactions in both preschool and school settings (9, 40, 41).

Furthermore, as suggested by Coplan and Weeks (2), language *skills* could be a positive resource for shy children’s socioemotional functioning. That is, shy children who are more able to communicate their thoughts and who are confident in their ability to use appropriate language may be less prone to feel anxious and wary around peers. Better language skills could also foster positive social interactions, and may, in this sense, be an essential tool that could aid shy children to make and keep friends. There is some support for this proposition from a handful of studies demonstrating that shy children who also are verbally skilled (i.e., expressive, receptive, and pragmatic language skills) tend to show less inhibition, loneliness, social anxiety, peer difficulties, and asocial behaviors compared to shy children who are less verbally skilled (2, 15, 42). Despite these research efforts, there is still a lack of longitudinal studies examining such pathways over time.

Moreover, relatively less is known about the role of shy children’s social interaction skills and behaviors for their future socio-emotional and communicative functioning. As suggested by Asendorpf (24) as well as the “*shy but getting by*” model by Coplan et al. (13), such skills and behaviors are likely to promote resiliency and foster positive development among shy children by aiding in the formation of positive interactions and relationships with peers and others. In this sense, positive social skills, such as play behaviors with peers, may increase shy children’s social engagement and their opportunities to learn and practice social and communicative skills, which ultimately could ameliorate their increased risk of language and internalizing problems.

There is some support for such assertions from studies showing that shy (i.e., anxious solitary) children with higher levels of social competencies (i.e., more agreeable) had higher quality peer relations and were better liked by peers compared to shy children with lower levels of social competencies (14, 16). Further, studies also show that shy children with higher levels of positive affect (i.e., positive facial expression, smiling) have fewer symptoms of social anxiety and display more sociability and advanced theory of mind compared to children with more negative affect (4, 7). Thus, considering these results, it is plausible to expect social competencies in the form of social play behaviors with peers to also influence the prospective link between early shyness and later adjustment and language functioning. To date, however, such interactions are yet to be explored empirically.

### 1.1. The present study

On this background, the present study aspires to (1) examine the long-term association between shyness in childhood (from age 18 months to five years) and later adjustment outcomes (language and internalizing difficulties) in school at ages eight and 11 years, and (2) explore the possible influence of early social play behaviors and language competencies as measured in preschool in these prospective links.

First, based on previous research (3, 35), we hypothesize that higher levels of early shyness will be positively associated with both language problems as well as with internalizing problems during the school years (at age eight and 11 years). However, in accordance with a previous meta-analysis (28), we expect that associations with anxiety symptoms will be stronger than associations with depressive symptoms.

Second, building on previous theorizing and empirical evidence (13, 15, 16, 24), we explore if differences in early social play behaviors and language competencies during the preschool years could influence the strength of these longitudinal associations. More specifically, we hypothesize that the associations between early shyness and later language and internalizing problems will decrease at higher levels of social play behaviors and language skills but increase at higher levels of language problems and lower levels of social play behavior with peers.

Finally, as previous research has indicated that social play behaviors and language competencies may act as mediators in the associations between shyness and adjustment outcomes (33, 43, 44), we also explored whether social play behaviors and language competencies would account for (i.e., mediate) the prospective associations between early shyness and later adjustment outcomes in the current study. In this sense, we hypothesize that higher shyness in childhood predicts less social play behaviors in preschool, which in turn, predict poorer language abilities and adjustment in school. Similarly, based on perspectives arguing that shy children’s poor language abilities may reflect a “performance deficit” (because shy children’s wariness and social reticence may inhibit their propensities to speak in social settings), we hypothesize that higher levels of childhood shyness predict poorer language competencies in preschool, which, in turn, will be prospectively associated with poorer adjustment and language competencies in school.

Previous research has also demonstrated gender differences in shyness, as well as in its associations with developmental outcomes (2, 45, 46), although such differences have not been consistently reported (47, 48). Studies also have shown parental socioeconomic factors to be associated with a variety of child outcomes (49, 50). For this reason, we included both gender and mother’s education level in all our analyses.

## 2. Materials and methods

### 2.1. Participants and procedure

The participants of this study represent a sub-cohort from the Norwegian Mother, Father, and Child Cohort Study (MoBa). The MoBa study is a prospective population-based pregnancy cohort study conducted by the Norwegian Institute of Public Health (51, 52). Participants of the MoBa were recruited from all over Norway from 1999 to 2008. The women consented to participation in 40.6% of the pregnancies. The MoBa now includes 114,500 children, 95,200 mothers, and 75,200 fathers. Pregnancy and birth records from the Medical Birth Registry of Norway (MBRN) are linked to the MoBa database (53).

The sub-cohort of the current study includes a total of 7,447 children (50.1% girls). This sample consists of children born between 2006 and 2009 with ECEC teacher rated questionnaire data at five years of age. For this sub-cohort, we also included mother-rated data from child age 18 months, three, five, and eight years, and primary school teacher-rated data at child age 11 years (response rate = 51%).

The ECEC teachers (response rate = 40%) were recruited from all over Norway over a three-years period through invitation from the participating mothers, meaning that the sample was spread across different geographical locations. We have ECEC center ID on most of these children (*n* = 5,773) from across 2,738 ECEC centers. Among these children, the majority (*n* = 3,035) were the sole target-child in the center, whereas the remaining children were in the same ECEC center as one or more children in the sample. Although we have no information about whether these children were in the same department or not, there is a possibility that some children might have been rated by the same ECEC teacher and, thus, there is a possibility of interdependence between observations. However, we have previously explored the potential of clustering effects at the ECEC level (i.e., multiple children in the same ECEC center) in the subsample and found that results remained identical, which suggests that the effect of clustering is limited (50). Due to missing ID information for the schools included, we were not able to test for clustering effects at the primary school level. Thus, we cannot rule out the possibility of interdependence between observations at the school level.

The establishment of MoBa and initial data collection was based on a license from the Norwegian Data Protection Agency and approval from The Regional Committees for Medical and Health Research Ethics. The MoBa cohort is regulated by the Norwegian Health Registry Act. Written informed consent was obtained from all participants. The present research project is approved by the Regional Committees for Medical and Health Research Ethics (REK) (2015/1324). We use the twelth version of the quality-assured dataset released for research in 2019 (54).

### 2.2. Measures

#### 2.2.1. Shyness

Mothers assessed child shyness at child age 18 months, three, and five years *via* the shyness subscale of the *Emotionality, Activity, and Sociability Temperament Survey–Short Form* [EAS; (55)]. Previous studies have demonstrated satisfactory psychometric properties for the shyness subscale (56). This subscale originally includes five items rated on a 5-point scale (from 1 = *not typical* to 5 = *very typical*), but only three questions were included for use in the MoBa questionnaire (i.e., “Is very social,” “Is very friendly with strangers,” both reversed, and “Takes a long time to warm up to strangers”). The Cronbach’s alpha for the shyness subscales was 0.65 at age 18 months, 0.67 at age three years, and 0.71 at age five years.

#### 2.2.2. Social play behaviors

At child age five years, ECEC teachers rated the target child’s play behaviors with peers using the social play subscale of the Preschool Play Behavior Scale [PPBS; (57)]. The subscale comprises five items that assess social play in terms of the extent to which the child engages in peer conversation (“Engages in active conversations with other children during play”) and in group interaction [e.g., “Plays in groups with (and not just beside) other children”], with response categories ranging from 1 = *never* to 5 = *very often*. The subscale is previously shown to display acceptable psychometric properties, including high internal reliability (α = 0.96) and good construct validity (57). The PPBS instrument has been translated and back-translated and used in several different cultures, including Italy (44), Finland (58), Korea (59), Norway (60), Malaysia (61), and Turkey (62). The internal reliability (α) for the social play subscale in the current study was 0.66.

#### 2.2.3. Language competencies

At child age five years, ECEC teachers rated the target child’s language competencies using a combination of items from two language subscales of the Child Development Inventory [CDI; (63)]. Five items were taken from the originally 50-items verbal comprehension subscale [i.e., “Tells where he(she) lives, naming town or city,” “Uses the words “today,” “yesterday,” and “tomorrow” correctly] while four items were taken from the originally 50-items expressive language subscale (i.e., “Asks the meaning of words,” “Uses irregular plurals correctly, for example, says “men,” not “mans”), with dichotomous response categories (1 = *no*, 2 = *yes*). Previous studies have demonstrated acceptable psychometric properties of the CDI, including internal reliability and construct and predictive validity (64). The polychoric reliability of the teacher-rated language competence at age five years was 0.69.

At child age eight years, mothers rated the child’s language competencies using the checklist of 20 statements about language difficulties (65). The checklist is a validated Norwegian instrument used to identify children with receptive, semantic, and expressive language difficulties. Eight of the original 20 items were selected for use in the MoBa (e.g., “Is often struggling finding the right words,” “Has difficulties understanding the meaning of common words”), with statements rated on a 5-point scale (from 1 = “*Does not fit the child/absolutely wrong*” to 5 = “*Fits well with the child, absolutely right*”). The polychoric reliability of the mother-rated and teacher-rated language competencies at age eight were 0.85 and 0.78, respectively.

At child age 11, school teachers rated the target child’s language competencies using the Children’s Communication Checklist [CCC-2; (66)]. The CCC-2 is a screening instrument used to identify language impairment in children that originally includes ten subscales with seven items each. Twelve items were selected for use in the teacher questionnaire, comprising word-finding difficulties, appropriate use of language, coherence, and syntax (e.g., “Forgets words s/he knows,” “Mixes up words of similar meanings,” “Can produce long and complicated sentences such as: When we went to the park I had a go on the swings;” “I saw this man standing on the corner,” “Uses terms like “he” or “it” without making it clear what s/he is talking about”) rated on a 5-point scale (from 1 = “*Does not fit the child/never*” to 5 = “*Fits well with the child/always*”). Previous studies have shown the CCC-2 to have good psychometric properties (67). The polychoric reliability of language competencies at age 11 years in this study was 0.84.

#### 2.2.4. Internalizing problems

Children’s anxiety symptoms were measured at age eight (mother reports) and at age 11 years (teacher reports) using the short version of the Screen for Child Anxiety Related Disorders [SCARED; (68)]. The SCARED is a multidimensional instrument intended to measure anxiety symptoms corresponding to DSM-defined anxiety disorders. The instrument originally contains 41 items covering five subscales, but the five-item short form was selected for use in the MoBa (i.e., “The child gets really frightened for no reason at all,” “People tell the child that he/she worries too much”), with response categories ranging from 1 = *Not true* to 3 = *True*. The short form is previously found to have good psychometric properties similar to the full version (69). The polychoric reliability of anxiety symptoms was 0.66 at age eight years and 0.67 at age 11 years.

Children’s depressive symptoms were measured at age eight (mother reports) and at age 11 (teacher report) using the Short Mood and Feelings Questionnaire [SMFQ; (70)]. The SMFQ consists of 13-items based on DSM-III-R criteria for depression, composed by descriptive phrases regarding how the individual has been feeling or acting recently (e.g., “Didn’t enjoy anything at all,” “Felt s/he was no good anymore”), with response categories ranging from 1 = *Not true* to 3 = *True*. The SMFQ is found to have good psychometric properties (71). The polychoric reliability of depressive symptoms was 0.79 at age eight years and 0.76 at age 11 years.

#### 2.2.5. Covariates

Gender and maternal education were included as covariates in the analyses. Gender was indexed using birth records of boys and girls (50.1%) from the Medical Birth Registry of Norway. Maternal education was measured using mother’s self-reported level of education derived from the MoBa 15th weeks of pregnancy questionnaire with response categories ranging from nine-years secondary school to University/College over four years. Due to the small number of participants in the lowest categories, the education variable was reduced to three categories scored as: (1) up to high school education (20.4%), (2) higher education college/university up to four years (44.5%), and (3) higher education college/university more than four years (35.1%).

### 2.3. Statistical analysis

All analyses were performed within a structural equation modeling framework using MPlus version 8.2 (72). Full information maximum likelihood with robust standard errors (MLR) was used to handle missing data and to correct test statistics and standard errors for non-normality of the observations (73). Model fit was evaluated by values of the Comparative Fit Index (CFI) and Tucker Lewis Index (TLI) above 0.95 and by Root Mean Square of Approximation (RMSEA) below 0.05 (74).

Analyses were carried out in several steps. First, measurement models for all study variables were estimated by using confirmatory factor analyses (CFA) whereby we constructed one latent factor for each of the variables based on their respective indicators. Second, we tested measurement invariance of shyness across the three time points by comparing a baseline model (i.e., configural invariance) against a series of increasingly restricted models (i.e., weak invariance and strong invariance), following Widaman et al. (75). In the configural invariance model, we estimated the first loading and the first intercept, while the corresponding first factor loadings and factor intercepts were constrained to be invariant over time. In the weak invariance model, we added across-time invariance constraints on the remaining factor loadings, and in the strong invariance model, across-time invariance constraints were also placed on the remaining factor intercepts. The model fit of the most restricted invariance model (i.e., strong invariance) was adequate [χ^2^(21) = 1145.01, RMSEA = 0.025 (95% CI: 0.024, 0.027), CFI = 0.99, TLI = 0.98, SRMR = 0.026] and not significantly worse than the less restricted models [Δχ^2^(2) = 16.71, *p* > 0.05].

This baseline measurement model was then used to construct a second order latent childhood shyness factor based on the three latent shyness factors. We established an approximate standard metric by constraining the first factor loading of the shyness factor to its specific value and by setting the mean of the latent shyness factor at the first time point to 0 and the variance of the factor to 1.

Third, we examined the prospective associations from the early childhood shyness latent factor as well as from the latent social play behavior and language competencies factors at age five years to later language and internalizing problems at age eight and age 11 years by using multiple regression analyses, controlling for gender and maternal education. For the outcomes at age eight years, all the preschool predictor variables were simultaneously included. For the outcomes at age 11 years, we estimated both univariate (including only the preschool predictor variables) and multivariate models (controlling for previous levels of the variable, for instance; anxiety at age eight for anxiety at age 11 years).

Fourth, we tested whether early social play behaviors and language competencies moderated associations of childhood shyness with language problems and internalizing problems by estimating a series of latent moderation structural equation models [LMS; (76)], one for each outcome variable at child age eight and 11 years. The LMS approach produces estimates of interaction that are not attenuated by measurement errors, which in turn serves to increase power as well as reducing the likelihood of biased estimates (77). In each model, the predictor (latent childhood shyness), the moderator in question (social play behavior, language competence), and the product term of these variables were simultaneously included as predictors of the outcome variables, while also controlling for gender and maternal education. Simple slopes follow-up analyses were then conducted to further probe the association between childhood shyness and the outcome variables at different levels of the moderator variables, specified to low (−1 SD below the mean), average, and high levels (+1 SD above the mean) of the moderator variables. Prior to all analyses, we standardized the latent variables by setting their variances to 1 and freeing their first indicator loadings. We also tested for gender differences in all analyses.

Finally, we estimated several indirect models for each of the mediator variables, social play behaviors and language competencies, where these variables were defined as putative mediators in the association between early shyness and each of the outcome variables at age eight and 11 years, respectively, controlling for gender and maternal education. For these models, we applied path analyses and estimated the indirect paths and their confidence intervals by using bootstrapping (78), and tested for gender differences by comparing constrained paths with unconstrained pats. In the indirect models for the outcome variables at age 11 years, we also controlled for previous levels of the variable in question.

## 3. Results

Descriptive statistics (i.e., means, standard deviations, and range) and polychoric (i.e., latent) intercorrelations among the study variables are presented in Table 1. The patterns of associations were mostly as expected. There were high intercorrelations between the three shyness measures across time points. Further, shyness across all three time points was positively correlated with both language problems and anxiety at age eight. Language competency at age five years was only negatively associated with shyness at age three years, and social play behaviors at age five years was only negatively associated with shyness at age five years. Shyness at both ages three and five years was positively correlated with depressive symptoms at age eight years and with anxiety at age 11 years.

**TABLE 1:** Polychoric intercorrelations between study variables and descriptive statistics of variables.

	1	2	3	4	5	6	7	8	9	10	11	*M* (*SD*)	Range
1. Shyness 1.5 years (m)												2.05 (0.64)	1–5
2. Shyness 3 years (m)	0.69**											2.21 (0.68)	1–5
3. Shyness 5 years (m)	0.50**	0.77**										2.09 (0.71)	1–5
4. Social PB 5 years (kt)	-0.01	-0.03	-0.05*									4.41 (0.56)	1–5
5. Lang. Comp. 5 years (kt)	-0.02	-0.05*	-0.02	0.39**								1.88 (16)	1–2
6. Lang. Prob. 8 years (m)	0.07*	0.11**	0.10**	-0.26**	-0.51**							1.32 (0.48)	1–5
7. Anxiety 8 years (m)	0.12**	0.21**	0.29**	-0.08*	-0.06*	0.24**						1.20 (0.24)	1–3
8. Depressive 8 years (m)	0.01	0.04*	0.07**	-0.08**	-0.09**	0.25**	0.45**					1.14 (0.19)	1–3
9. Lang. Prob. 11 years (t)	0.00	0.02	0.01	-0.13**	-0.26**	0.25**	0.04	0.09**				1.37 (0.43)	1–4
10. Anxiety 11 years (t)	0.04	0.09**	0.13**	-0.10*	-0.12**	0.19**	0.45**	0.22**	0.15**			1.13 (0.22)	1–3
11. Depressive 11 years (t)	-0.04	-0.01	0.01	-0.07*	-0.10*	0.09**	0.20**	0.20**	0.12**	0.47**		1.13 (0.28)	1–3
12. Gender	0.11**	0.07*	0.02	0.18**	0.09**	-0.07**	0.05*	-0.01	-0.07**	0.04	-0.06*		
13. Mother education	-0.03*	-0.02	0.00	0.04	0.08**	-0.07**	-0.03	-0.06**	-0.05*	-0.05*	-0.02		

All variables are latent factors except gender (girls = 1) and mother education, Social PB = social play behavior; Lang. Comp. = language competencies; Lang. Prob. = language problems; (m) = mother reports; (kt) = ECEC teacher reports; (t) = school-teacher reports; *M* = mean scores; *SD* = standard deviation, *p < 0.05, **p < 0.01.

Social play behavior was positively associated with language competencies and negatively associated with anxiety and depressive symptoms at both time points. Language competence at all time points was negatively correlated with anxiety and depressive symptoms at both age eight and 11 years. Gender (i.e., being a girl) was positively correlated with shyness at age 18 months and age three years, social play behaviors at age five, language competence at all time points, and with anxiety at age eight. Higher mother education was positively correlated with language competence at all time points and negatively correlated with depressive symptoms at age eight years.

### 3.1. Direct path analyses

Concerning the outcome variables at age eight years (see Table 2), results from the multiple regression analyses revealed that childhood shyness was positively and significantly predictive of language problems and of anxiety and depressive symptoms. Further, ECEC teacher-reported social play behavior at age five years was negatively predictive of mother-reported language and internalizing difficulties, whereas ECEC teacher-reported language competency in preschool was only negatively predictive of later language problems as reported by mothers.

**TABLE 2:** Results from univariate and multivariate regression analysis with childhood shyness and social play behaviors and language competencies at age 5 years as predictors of internalizing and language problems at age 8 and 11 years.

Predictors	Childhood shyness age 1.5–5	Social play behaviors age 5	Language competencies age 5	Language problems age 8	Anxiety symptoms age 8	Depressive symptoms age 8
	**β (SE)**	**β (SE)**	**β (SE)**	**β (SE)**	**β (SE)**	**β (SE)**
**Outcomes age 8 years**						
Language problems	0.09** (0.02)	−0.06* (0.02)	−0.48** (0.02)			
Anxiety symptoms	0.24** (0.03)	−0.07* (0.03)	−0.04 (0.03)			
Depressive symptoms	0.05* (0.02)	−0.05* (0.02)	−0.05 (0.03)			
**Outcomes age 11 years**	*Univariate analyses*					
Language problems	0.00 (0.02)	−0.02 (0.03)	−0.24** (0.04)			
Anxiety symptoms	0.09** (0.03)	−0.09* (0.04)	−0.09* (0.04)			
Depressive symptoms	−0.02 (0.03)	−0.03 (0.04)	−0.09* (0.04)			
	*Multivariate analyses*					
Language problems	−0.01 (0.02)	−0.01 (0.03)	−0.15** (0.04)	0.17** (0.03)	−0.02 (0.03)	0.03 (0.03)
Anxiety symptoms	−0.00 (0.03)	−0.05 (0.04)	−0.04 (0.05)	0.06 (0.04)	0.42** (0.04)	−0.00 (0.03)
Depressive symptoms	−0.05 (0.03)	−0.02 (0.04)	−0.08 (0.05)	−0.02 (0.04)	0.14** (0.04)	0.13** (0.03)

*p < 0.05, **p < 0.001. All analyses controlled for gender and maternal education.

With regards to the outcome variables at age 11 years, results from the univariate analyses showed mother-rated childhood shyness and ECEC teacher-reported social play behaviors and language competencies in preschool to be significantly predictive of teacher-reported anxiety symptoms, and early language competencies in preschool to be negatively predictive of teacher-reported depressive symptoms. However, these associations were no longer significant when we included previous levels of the outcome variables at age eight in the analyses (see Table 2).

### 3.2. Moderation analyses

**FIGURE 1:**
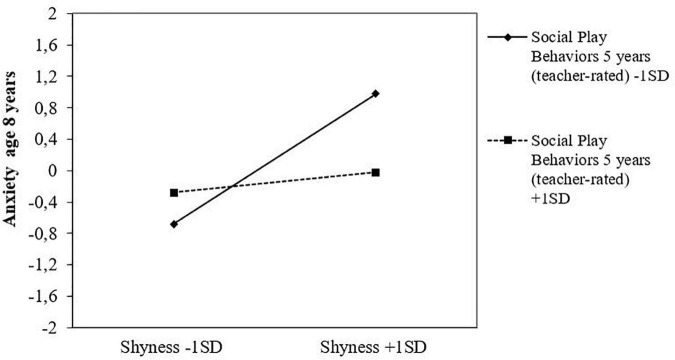
Social play behaviors at age 5 years as a protective factor in the association between childhood shyness and symptoms of anxiety at age 8 years, as reported in standard deviations.

**FIGURE 2:**
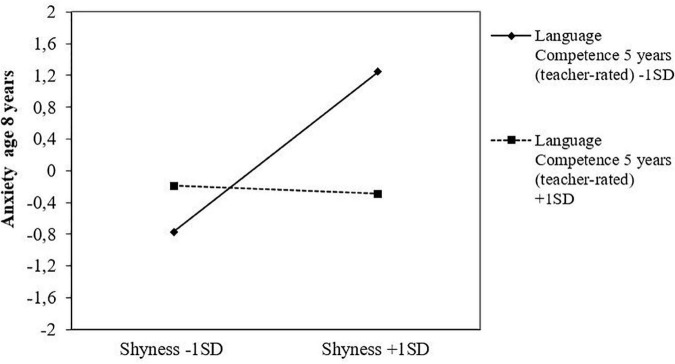
Language competencies at age 5 years as a protective factor in the association between childhood shyness and anxiety symptoms at age eight, as reported in standard deviations.

Results from the latent moderation analyses showed a few significant interaction effects. First, the childhood shyness x social play behavior interaction effect was significant for mother-rated anxiety symptoms at age eight. The negative coefficient of the interaction term (*b* = −0.09, *p* < 0.001) indicates that the otherwise positive association between childhood shyness and later anxiety in school-age decreased at higher levels of social play behaviors in preschool. Second, there was also a significant interaction effect of childhood shyness x language competencies for anxiety symptoms at age eight, where the negative coefficient of the interaction term (*b* = −0.14, *p* < 0.001) indicates that the positive association between shyness and anxiety decreased at higher levels of language competencies. Results from simple slopes further confirmed this, by showing that the association with anxiety symptoms steadily decreased at higher levels of the two moderator variables (see Table 3). Figures 1, 2 illustrate the graphical plot of these interaction effects, demonstrating that when early social play behavior increased by one unit, the association between shyness and anxiety became less strong, decreasing by 0.09 standard deviations (Figure 1). Similarly, when language competencies increased by one unit, the association between shyness and anxiety decreased by 0.14 standard deviations (Figure 2). No interaction effects of early social play behaviors or language competencies in preschool were found for the associations between childhood shyness and the other outcome variables (i.e., depressive symptoms and language problems).

**TABLE 3:** Results from interaction analyses including tests of simple slopes of interaction of the moderators social play behaviors and language competencies with 95% confidence intervals.

	Anxiety symptoms age 8 years		
	***b***	**[95% CI]**	**β (SE)**
**Model 1: Social play behaviors**			
Childhood shyness (*x*)	0.12**	[0.098, 0.141]	0.24** (0.04)
Social play behaviors (*w*)	−0.04**	[−0.054, −0.019]	−0.07** (0.03)
Shyness*Social play behaviors (*xw*)	−0.09**	[−0.105, −0.068]	−0.12** (0.04)
**Simple slopes**			
−1 *SD* below the mean	0.21**	[0.171, 0.241]	
Mean	0.12**	[0.098, 0.141]	
+1 *SD* above the mean	0.03**	[0.014, 0.052]	
**Model 2: Language competence**			
Childhood shyness (*x*)	0.11**	[0.082, 0.135]	0.24** (0.05)
Language competencies (*w*)	−0.06**	[−0.091, −0.037]	−0.09** (0.05)
Shyness*Language competencies (*xw*)	−0.14**	[−0.173, −0.113]	−0.16** (0.05)
**Simple slopes**			
−1 *SD* below the mean	0.25**	[0.205, 0.299]	
Mean	0.11**	[0.082, 0.135]	
+1 *SD* above the mean	−0.03*	[−0.066, −0.002]	

x = predictor variable; w = moderator variable; xw = interaction term; 95% CI = 95% confidence interval; SE = standard error; *p < 0.05, **p < 0.01. All analyses controlled for gender and maternal education.

### 3.3. Indirect path analyses

Results showed no evidence of indirect effects of social play behaviors or language competencies in preschool in the links from childhood shyness to the three outcome variables at age eight and 11 years. However, results did indicate stability in the indirect pathways from childhood shyness to each of the outcome variables throughout childhood. More specifically, there was a significant indirect path from childhood shyness to teacher-rated anxiety symptoms at age 11 years through mother-reported anxiety symptoms at age eight [β = 0.103, 95% CI (0.072, 0.145), *p* < 0.001]; from childhood shyness to teacher-rated depressive symptoms at age 11 years through mother-rated depressive symptoms at age eight [β = 0.009, 95% CI (0.003, 0.018), *p* < 0.001]; and from childhood shyness to teacher-rated language problems at age 11 years through mother-rated language problems at age eight [β = 0.016, 95% CI (0.009, 0.021), *p* < 0.001].

## 4. Discussion

The overall scope of the current study was to explore the long-term association between shyness in childhood and later adjustment outcomes (language and internalizing difficulties) in school, and secondly, to explore the role of early social play behaviors and language competencies in preschool in these prospective links.

Among the results, a higher level of shyness in childhood was prospectively predictive of later language problems and symptoms of anxiety and depression in early school age. Second, results showed higher levels of social play behaviors and language competencies in preschool to serve a protective role for shy children’s risk of later internalizing difficulties by significantly moderating (i.e., reducing) the prospective associations from childhood shyness to anxiety symptoms at age eight. In contrast, there were no indications that early social play behaviors and language competencies accounted for (i.e., mediated) the prospective links from early shyness to later adjustment difficulties. A closer elaboration of the results and their implications follow below.

### 4.1. The emotional functioning of shy children

In this study, results revealed that childhood shyness, as measured across 18 months, and three and five years, predicted symptoms of anxiety and depression later in childhood, both directly at age eight (mother-ratings), and indirectly at age 11 (teacher-ratings) through previous symptom levels at age eight. In this sense, the results are suggestive of some degree of stability in the longitudinal pathways from early shyness to later internalizing difficulties throughout the childhood years. However, importantly, the significant association between shyness and later depressive symptoms was weaker than the longitudinal association between shyness and later anxiety symptoms, indicating that shyness represents a greater risk factor for anxiety more than for depression in childhood. This finding aligns with previous studies reporting stronger associations of shyness with anxiety, particularly social anxiety, than other internalizing difficulties (3, 28, 32, 79). In other words, the results of our study bring some support to the notion that temperamental shyness in childhood, characterized by excessive wariness in novel social contexts and with new people, may represent one of the strongest risk factors of anxiety in the subsequent development phases (80). Furthermore, the age period considered in the current study (i.e., school-age) might be an early stage for developing depressive problems, which may emerge in the following developmental periods (3). Thus, identifying shy children at risk in the early phases of development could be helpful to reduce the risk for later internalizing problems, especially concerning anxiety symptoms.

### 4.2. The socio-communicative functioning of shy children

With respect to language problems, our results corroborated with previous research findings demonstrating shy children’s increased risk of language difficulties, including poorer pragmatic, expressive and receptive language abilities (2).

However, there has been less agreement about the extent to which such difficulties may reflect a “performance deficit,” that is, whereby shy children’s fearful and anxious demeanor may directly inhibit their propensity to speak in social situations (81, 82), or whether such difficulties may rather reflect a “competence deficit,” whereby shy children’s withdrawn and restricted participation in social interaction may influence these children’s opportunities to learn and practice language skills (83). In the former case, one would expect language difficulties to account for (i.e., mediate) the links between shyness and poor adjustment, whereas in the latter case, one would expect language difficulties to influence (i.e., moderate) the strength of such associations.

In many respects, the results of the current study add important knowledge to this ongoing debate. While there was no evidence of mediation effects of language skills for any of the prospective links, the results showed language competencies in preschool to moderate the longitudinal links between early shyness and later anxiety symptoms. More specifically, this moderating effect suggests that shyness and language skills represent two separate characteristics of the child, which, when co-occurring as high shyness and low language skills, may jointly put the child at risk of anxiety symptoms in the long run. However, when high shyness is accompanied by high language skills, shy children’s risk of later anxiety may decrease significantly. In this sense, our results primarily provide support for the view that language abilities may largely reflect a “competence deficit” rather than a “performance deficit.” Thus, bearing in mind the crucial impact of language skills for shy children’s adjustment in several developmental areas (2, 15, 42), our findings may have important implication for prevention purposes insofar as they highlight the potentially positive benefits of targeting shy children’s language abilities at an early age. Importantly, these results also indicate that language difficulty is not necessarily a defining feature of all shy children, but rather suggest that heterogeneity in shy children’s language abilities is likely to depend on the influence of other individual and/or environmental factors.

### 4.3. The social functioning of shy children

Additionally, the current study also demonstrated the protective benefits of early social play behaviors with peers for shy children’s later adjustment. More specifically, the association between childhood shyness and symptoms of anxiety at age eight was stronger at lower levels of social play behaviors but was found to decrease significantly at higher levels of social play behaviors. Previous research has, indeed, confirmed the benefits of positive social actions for experiencing positive emotions, sense of belonging, and overall well-being (84, 85). As such, this result adds to the growing body of research showing positive social behaviors to buffer against negative adjustment outcomes for shy children, for instance such as peer problems (14, 16) and social anxiety (4, 7).

Drawing on previous suggestions, a possible mechanism behind such links could be that positive social behaviors and competencies are likely to facilitate more peer liking and more positive interactions with others, which ultimately may help reduce shy children’s risk of socioemotional problems (4). As such, shy children who practice social behaviors and skills may gradually familiarize themselves with others and, consequently, feel better and less socially wary and anxious in social situations. In this sense, the findings of our study add to the growing literature suggesting that when shyness is accompanied with positive behaviors or characteristics, it becomes less strongly associated with anxiety.

### 4.4. Gender differences

Although gender correlated significantly with several of the study variables, we did not find any significant gender differences in any of the associations between the study variables. In past research, there have been contradictory findings concerning this issue, with some studies reporting stronger associations between shyness and internalizing symptoms among boys relative to girls (45), while others find that such associations are stronger for girls than for boys or that there are no gender differences at all (86, 87). Potential reasons for this inconsistency in the findings might be due to differences in *how* shyness is operationalized and measured (i.e., as social withdrawal, conflicted shyness, or social disinterest) across different studies, as well as in *when* assessments are made (i.e., early vs. later childhood). For instance, although studies often show that there are no gender differences in shyness overall, there are indications that girls tend to “over-report” their shyness in middle and later childhood (86), which then could mask potential gender differences in associations with outcomes (88). Accordingly, further research into age-specific gender differences in the longitudinal associations between shyness (including different types of shyness) and developmental outcomes (i.e., social anxiety, loneliness) is clearly warranted.

## 5. Limitations

Despite the benefits of a longitudinal design, large sample size, and performing the analyses within a SEM framework, the present study also has limitations.

First, the correlational nature of this study precludes us from drawing conclusions with respect to causality and direction of associations. For instance, it has been suggested that the direction of influence may not only flow from shyness to poor language, but that lower language abilities may also lead to shyness (2, 35). However, there is limited support for this suggestion with research showing that such patterns may mainly exist among boys but not girls (36).

Second, we measured shyness by using the short form of the EAS which only includes three of the original five items. This may be problematic with respect to possible discrepancies in how shyness was operationalized and measured in this study. Yet, previous research has shown the short form to have satisfactory psychometric properties, including reliability and validity estimates approaching those of the original scale and with high correlations (*r* = 0.95) observed between the short-form and the original form (89). As such, these similarities may indicate that the short form, despite its limited number of items, is sufficient in terms of capturing the most essential and core features of the shyness phenomenon among children and youth.

Third, the measure of anxiety symptoms in the current study taps general anxiety, whereas there is evidence to suggest that shyness is most strongly associated with social anxiety (28). This may partly explain why the direct association between shyness and anxiety at age 11 years was not significant. Thus, future studies should examine longitudinal associations between early shyness and specific types of anxiety symptoms across different phases of development.

Fourth, the effect sizes for the associations between the study variables were small to moderate, and this warrants that our results should be interpreted with caution. The small effect sizes are problematic because although we demonstrate statistically significant results, this does not necessarily mean that they have practical significance, which is an important issue to consider with regards to intervention purposes. However, given that the assessment points expanded over a long period of time, we did not expect to see large effect sizes in this study. It is possible that the vast number of idiosyncratic experiences occurring within these formative child years may have contributed to the children’s socioemotional adjustment and language development above and beyond the contribution from the included variables in this study. Another possibility is that the over-representation of well-functioning and well-educated families in this study compared to the population in general (90), might have led to an underestimation of true effect sizes.

Fifth, the validity and generalizability of the study findings may be restricted by general limitations of the MoBa sample, such as attrition, selection, and non-response bias. For instance, problems with self-selection bias and attrition may have resulted in biased estimates of the associations and underestimation of effect sizes (90). Recent research suggests using multiple imputation or inverse probability weighting to account for selection bias present in the MoBa cohort due to loss to follow-up (91). However, others question the appropriateness of using such imputation techniques uncritically as not all associations necessarily are impacted by selection bias (92). In the current study, we applied full information maximum likelihood with robust estimators (MLR) to handle missing data, following recommendations of Lodder et al. (73).

Sixth, we had a valid ECEC center ID for around 78% of the sample but no equivalent ID at the school level in the study. Thus, we cannot rule out the possibility of interdependence between the observations in cases where the same teachers reported on more than one target child. However, the issue on non-independence in observations should not be a substantial concern as the children participating in the MoBa are dispersed across different ECEC centers and schools all over Norway. Furthermore, we have in previous research using the same subsample shown that results remained the same when we adjusted standard errors for clustering at the ECEC level to allow for interdependence in the observations (50). Together, these aspects suggest that the effect of clustering is rather limited.

Finally, as our investigation involved Norwegian children, their mothers, and teachers, the generalizability of the findings may be limited to Norwegian contexts.

## 6. Conclusion

In sum, the findings of the current study address some of the shortages in the literature concerning which early behavioral child characteristics that may influence the strength and direction of the prospective associations between childhood shyness and later internalizing and language problems. Our results provide empirical support to the theoretical propositions that positive behavioral features, such as social play behaviors and language competencies, may be particularly adaptive for shy children’s socio-emotional functioning and adjustment (13). As such, this study adds to the emerging body of research demonstrating the importance of exploring how traits may operate in an interactive manner in influencing shy children’s developmental outcomes (6, 93). In this sense, the present study offers novel input for both developmental and child personality research by providing evidence in support of a more heterogeneous and nuanced conceptualization of the shyness dimension. Most importantly, the above findings offer some optimistic implications for shy children by showing that positive behavioral assets, such as social play behaviors and language competencies, may buffer against these children’s increased risk of internalizing problems. Future intervention training programs should aim to reinforce shy children’s social behavior and communication skills, both to facilitate and aid their desire to interact and being accepted by others, but also to reduce their inhibited and withdrawn behaviors and thereby their subsequent risk for internalizing difficulties.

## Data availability statement

The original contributions presented in this study are included in this article/supplementary material, further inquiries can be directed to the corresponding author.

## Author contributions

All authors listed have made a substantial, direct, and intellectual contribution to the work, and approved it for publication.
